# Improving the Quality of Follow-Up Documentation Using a Structured Subjective, Objective, Assessment, and Plan (SOAP)-Based Template: A Two-Cycle Clinical Audit at Hasahesa Teaching Hospital, Sudan

**DOI:** 10.7759/cureus.100189

**Published:** 2025-12-27

**Authors:** Maram Hassan Ali Mohamed, Mohamed Hassan Ali Mohamed, Amro Osama Fathi Mohammed, Maisoon Musa, Abd Elazim Mohamed, Somia Elhag, Sara Elhadi, Fatima Ahmed, Fatima Dahab, Hibatalla Mohammed, Azza Abdelrahman Abdallah Ahmed, Alshanna Mohamed Saeed Mohamed Ahmed, Eithar Hassan Mahgoub Babiker, Khattab Mohammed Aboudi Ahamed, Basma Babiker, Obada Alwaleed Ahmed Mohamed, Yahya Mahmoud Abozamr, Entisar Abdelfarag Ahmed Fadlemola, Altayib Abdalrahman, Maram A Babikir, Nassma Ibrahim

**Affiliations:** 1 Department of Neonatology, Maternity and Children's Hospital - Najran, Najran, SAU; 2 Department of Surgery, Al Neelain University, Khartoum, SDN; 3 Department of Internal Medicine, Al Neelain University, Khartoum, SDN; 4 Department of General Practice, Al Neelain University, Khartoum, SDN; 5 Department of Emergency, Hasahesa Teaching Hospital, Al‑Hasahisa, SDN; 6 Department of General Practice, Hasahesa Teaching Hospital, Al‑Hasahisa, SDN

**Keywords:** clinical audit, documentation improvement, follow-up notes, hasahesa teaching hospital, internal medicine, medical record quality, quality improvement, soap documentation, sudan

## Abstract

Background

Accurate and well-structured follow-up documentation is essential for effective clinical decision-making, continuity of care, and patient safety. In many resource-limited, paper-based hospital settings, follow-up notes are often incomplete or inconsistently structured, resulting in communication gaps and suboptimal care. This audit evaluated whether introducing a standardized Subjective, Objective, Assessment, and Plan (SOAP)-based template, supported by targeted staff training, could improve the completeness and organization of follow-up notes in the Internal Medicine Department at Hasahesa Teaching Hospital in Khartoum, Sudan.

Methods

A prospective two-cycle clinical audit was conducted over six months. Follow-up notes were assessed using a structured proforma based on the SOAP format. In the first cycle, 53 notes were reviewed, and 50 in the second. Between cycles, a multifaceted intervention was implemented, consisting of a standardized SOAP documentation template, staff education sessions, and routine reinforcement. Data were analyzed descriptively, and pre- and post-intervention differences were evaluated using chi-square testing.

Results

Marked improvements were observed across all SOAP domains following the intervention. Documentation of key subjective elements - including presenting complaint, past medical history, and review of systems - showed substantial gains. Objective documentation improved through more consistent recording of physical examinations and diagnostic results. Clinical reasoning was more clearly articulated through improved recording of primary and differential diagnoses, while planning elements, such as investigations, treatment updates, referrals, and patient education, also demonstrated strong improvement. Overall, adherence to the SOAP structure rose considerably after the intervention.

Conclusion

Introducing a structured SOAP-based template, reinforced by targeted training, significantly enhanced the completeness and organization of follow-up documentation in a paper-based, resource-constrained setting. The intervention proved simple, practical, and highly effective for improving documentation quality. Sustaining these gains will require ongoing education, periodic re-audits, and the integration of structured documentation expectations into departmental practice. Further research is needed to evaluate the impact of improved documentation on clinical outcomes and patient safety.

## Introduction

Clinical documentation is a cornerstone of safe and effective patient care, particularly in inpatient internal medicine settings, where follow-up notes provide continuity between successive clinicians. These notes function not only as a record of clinical progress but also as a handover tool that guides ongoing assessment, decision-making, and treatment planning. Deficiencies in documentation - such as incomplete entries, poor organization, and the absence of clear management plans - have been widely reported across healthcare systems and are associated with compromised continuity of care and an increased risk of adverse events [1]. Ensuring the quality and consistency of follow-up notes is therefore essential for maintaining patient safety and professional accountability.

Structured documentation formats have been proposed as practical solutions to improve the clarity and completeness of clinical notes. One of the most widely recognized approaches is the Subjective, Objective, Assessment, and Plan (SOAP) format, which originates from the problem-oriented medical record model [2]. By organizing clinical information into distinct and logical sections, the SOAP format supports clinical reasoning, facilitates communication among multidisciplinary teams, and enhances the readability of medical records [3]. In the context of inpatient follow-up notes, this structure encourages explicit documentation of patient-reported symptoms, objective clinical findings, diagnostic impressions, and planned interventions.

Despite these recognized advantages, audits of real-world practice consistently demonstrate suboptimal adherence to structured documentation principles. Studies involving medical trainees and junior doctors have identified frequent omissions in key SOAP components, particularly within objective findings and management plans [4]. Quality improvement studies using audit-and-feedback methodologies have shown that targeted interventions - such as focused education, standardized templates, and regular reinforcement - can lead to meaningful improvements in documentation practices [5]. These findings highlight both the persistence of documentation gaps and the potential for structured, low-cost interventions to address them.

Documentation challenges are often more pronounced in low- and middle-income countries, where high patient volumes, workforce constraints, and reliance on paper-based records place additional pressure on clinicians [6]. In such settings, simple and standardized documentation tools that do not depend on electronic health record systems may offer a particularly effective and sustainable solution. Evaluating the impact of structured documentation within these contexts is therefore of practical importance and may yield transferable lessons for similar resource-limited healthcare environments.

Accordingly, this two-cycle clinical audit was conducted in the Internal Medicine Department of Hasahesa Teaching Hospital, Al‑Hasahisa, Sudan, to evaluate the quality of inpatient follow-up notes before and after the introduction of a SOAP-based documentation template supported by targeted staff training. In each audit cycle, 50 follow-up notes were reviewed over a six-month period.

The primary objective of this audit was to assess whether implementing a structured SOAP-based documentation template, combined with a focused educational intervention, improved the completeness and organization of inpatient follow-up notes. The secondary objectives were to identify baseline documentation deficiencies, compare documentation quality across individual SOAP components before and after the intervention, and assess the feasibility of this low-cost quality improvement approach in a resource-limited internal medicine setting.

## Materials and methods

This project was conducted as a prospective, two-cycle clinical audit aimed at evaluating and improving the quality of follow-up note documentation within the Internal Medicine Department of Hasahesa Teaching Hospital, Al‑Hasahisa, Sudan. The hospital is a busy, secondary-level teaching institution that receives referrals from surrounding rural areas and manages a diverse inpatient population with high turnover. In such settings, follow-up notes serve as the main vehicle for clinical communication between rotating teams; therefore, assessing and improving documentation quality is an essential patient-safety priority.

The audit was designed according to the standard Plan-Do-Study-Act (PDSA) and closed-loop audit methodology, ensuring systematic identification of gaps, implementation of a structured intervention, and objective reassessment. The total audit duration was six months, beginning in May 2024. Cycle 1 was conducted over a two-week interval and focused on assessing baseline documentation practices in routine follow-up notes. Following this, a two-month intervention phase was implemented to introduce and consolidate structured documentation practices. Cycle 2 extended over three months to ensure that the effects of the intervention could be reliably measured over a sustained period.

A total of 103 follow-up notes were reviewed - 53 in the first cycle and 50 in the second - selected using simple random sampling from daily inpatient medical charts. Notes were eligible for inclusion if they were written as part of routine internal medicine ward rounds for inpatients aged ≥18 years. Admission notes, discharge notes, and notes written by visiting specialty teams were excluded to maintain homogeneity and ensure the evaluation of typical daily follow-up entries only.

**Figure 1:**
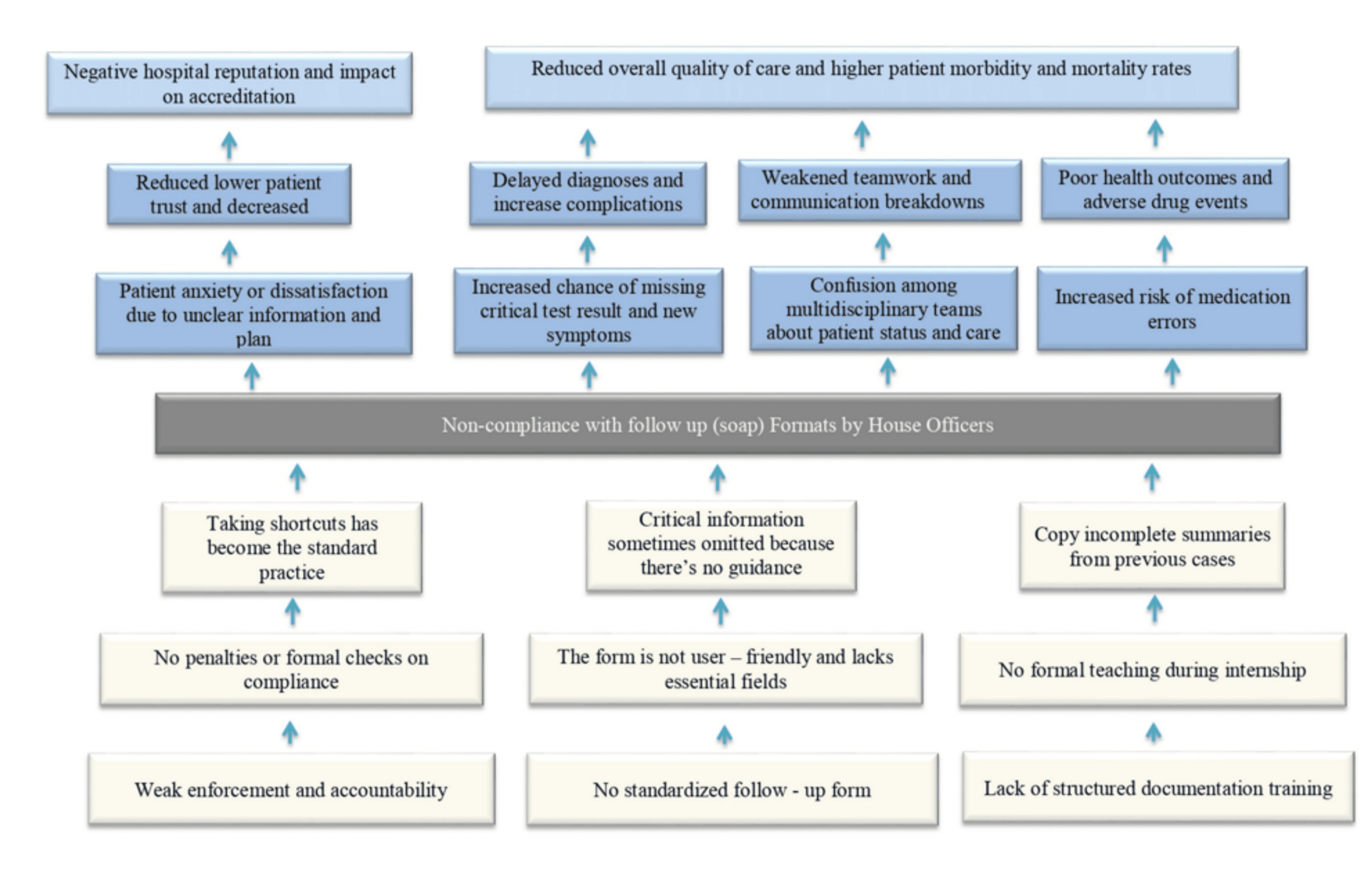
Problem Tree Analysis of Follow-Up Note Documentation Noncompliance This figure represents the authors’ original problem tree, illustrating the hierarchical relationships between the root causes, intermediate mechanisms, and clinical consequences of non-compliance with structured Subjective, Objective, Assessment, and Plan (SOAP) follow-up documentation among house officers. The root-level factors include weak enforcement, absence of a standardized note format, and lack of formal documentation training. These foundational issues give rise to maladaptive practices, such as shortcut-taking, omission of essential clinical details, and copying incomplete information from previous notes. These behaviors contribute to confusion among multidisciplinary teams, missed updates, delayed diagnoses, and increased risk of medication errors. At the system level, these effects culminate in reduced quality of care, patient dissatisfaction, adverse outcomes, and a negative institutional reputation, which may impact accreditation. This analysis informed the design of the intervention by addressing underlying drivers rather than superficial manifestations.

In order to systematically identify the root causes of poor documentation, a structured problem-tree analysis was undertaken during the preparatory phase of the audit. This tool enabled the research team to visualize the central problem, its direct drivers, and underlying system-level determinants, thus avoiding superficial or symptom-focused interventions. The analysis highlighted recurrent documentation omissions, lack of standardized note structure, inconsistent supervisory oversight, high clinical workload, and the absence of continuous feedback mechanisms as key proximal factors. Deeper root causes included limited awareness of best practices for clinical communication, insufficient orientation for new medical officers and interns, and variability in documentation expectations across rotating teams. The resulting problem tree was used to map causal pathways and informed the intervention design by ensuring that proposed solutions directly targeted these root factors rather than isolated manifestations (Figure 1).

A structured audit proforma was developed in alignment with the SOAP documentation framework to evaluate the quality of follow-up notes. Each entry was reviewed for the presence, clarity, and completeness of the four key SOAP components: the Subjective domain, capturing interval history and patient-reported symptoms; the Objective domain, documenting vital signs, physical examination findings, and other measurable clinical data; the Assessment domain, reflecting the clinician’s diagnostic impression or evaluation of the patient's progress; and the Plan domain, outlining proposed investigations, treatment adjustments, monitoring strategies, referrals, and follow-up arrangements. Follow-up notes were classified as complete when all four components were adequately recorded, and as incomplete when one or more elements were missing or insufficiently documented. No numerical scoring system was applied, as the audit focused on overall completeness and consistency rather than graded scoring, ensuring that the assessment process remained simple, reproducible, and aligned with routine clinical practice.

Between the two cycles, a multifaceted intervention package was implemented. This included the introduction of a redesigned, standardized SOAP-based follow-up note template incorporated into all inpatient charts. Formal educational sessions were conducted for interns and medical officers, focusing on the principles of structured documentation, the rationale behind SOAP, and examples of high-quality entries. Visual reminder posters were displayed in doctors’ rooms, ward corridors, and nursing stations. Senior physicians reinforced correct documentation practices during daily ward rounds. Additionally, weekly mini-audits were performed to monitor early adherence, with informal feedback delivered to encourage continuous improvement. This combined approach was intended to enhance awareness, increase consistency, and reduce variability in documentation practices.

Data extraction was conducted manually by two trained audit team members to minimize transcription errors. Data were entered into a secure spreadsheet and analyzed using descriptive and inferential statistics. Frequencies and percentages were used to compare documentation completeness between Cycle 1 and Cycle 2. Pre- and post-intervention differences were evaluated using chi-square tests, and a p-value of <0.05 was considered statistically significant. Inferential analysis was performed to quantify the magnitude of improvement following the quality improvement intervention, rather than to test causal hypotheses.

This project was classified as a service-evaluation and quality-improvement activity involving retrospective review of anonymized documentation, without patient involvement or modification of clinical management. Therefore, it met the criteria for exemption from full institutional review board review. Ethical approval was granted by the Hasahesa Teaching Hospital Ethics and Audit Committee (Approval No. HTH-QA-2024-017). All collected information remained confidential, anonymized, and securely stored in accordance with institutional standards and national guidelines for clinical audit conduct.

## Results

A total of 103 follow-up notes were reviewed across the two audit cycles, including 53 notes in Cycle 1 and 50 notes in Cycle 2. Substantial improvements were observed in the completeness and structure of documentation following the implementation of the SOAP-based intervention.

In the Subjective domain, documentation of the chief complaint increased from 28 entries (52.8%) in Cycle 1 to full compliance in Cycle 2 (100%). Similar improvements were seen in recording past medical history, rising from 18 entries (34.0%) to 46 entries (92.0%), and in documenting the review of systems, which increased from 12 entries (22.6%) to 44 entries (88.0%). Inclusion of current medications and allergies improved markedly, from 10 entries (18.9%) to 45 entries (90.0%). All improvements in the Subjective section were statistically significant (p < 0.001).

Within the Objective domain, documentation of vital signs was already high at baseline (94.3%) and reached full compliance (100%) after the intervention, although this change was not statistically significant (p = 0.262). More notable improvements were seen in physical examination findings, which increased from 25 notes (47.2%) to 48 notes (96.0%), and in the inclusion of laboratory or imaging results, which rose from 17 notes (32.1%) to 44 notes (89.0%). Both showed statistically significant changes (p < 0.001).

In the Assessment domain, documentation of the primary diagnosis improved from 26 entries (49.1%) to 50 (100%), while documentation of differential diagnoses increased sharply from 6 entries (11.3%) to 42 (85.0%). These changes were highly significant (p < 0.001), reflecting greater transparency in clinical reasoning after the intervention.

The Plan domain also showed great improvements. Clear plans for investigations increased from 23 entries (43.4%) to 49 (98.0%). Treatment or medication plans improved from 38 (71.7%) to 50 (100%). Documentation of referrals rose from 9 (17.0%) to 44 (87.0%), and patient counseling increased from 8 (15.1%) to 42 (85.0%). All enhancements within the Plan section were statistically significant (p < 0.001).

Overall, adherence to the correct SOAP sequence (S → O → A → P) rose from 18 notes (34.0%) in Cycle 1 to 47 notes (94.0%) in Cycle 2 (p < 0.001), demonstrating a substantial improvement in structured, consistent documentation following the intervention (Table 1).

**Table 1:** Comparison of Documentation Completeness Between Cycle 1 (n = 53) and Cycle 2 (n = 50) Following Implementation of a SOAP-Based Intervention The table presents the frequency and percentage of follow-up notes meeting each documentation criterion during Cycle 1 and Cycle 2. Chi-square testing was used to compare proportions before and after the intervention. Significant improvements across most parameters reflect enhanced adherence to the SOAP format, following staff training, template standardization, and reinforcement measures. A p-value < 0.05 was considered statistically significant. SOAP: Subjective, Objective, Assessment, and Plan

Domain	Indicator	Cycle 1 (n = 53)	Cycle 2 (n = 50)	Chi-square	p-value
Subjective	Chief Complaint	28 (52.8%)	50 (100.0%)	28.631	p < 0.001
	Past Medical History	18 (34.0%)	46 (92.0%)	34.409	p < 0.001
	Review of Systems	12 (22.6%)	44 (88.0%)	41.704	p < 0.001
	Medications/Allergies	10 (18.9%)	45 (90.0%)	49.494	p < 0.001
Objective	Vital Signs	50 (94.3%)	50 (100.0%)	1.257	p = 0.262
	Physical Examination	25 (47.2%)	48 (96.0%)	27.399	p < 0.001
	Lab/Imaging Results	17 (32.1%)	44 (89.0%)	31.045	p < 0.001
Assessment	Primary Diagnosis	26 (49.1%)	50 (100.0%)	31.937	p < 0.001
	Differential Diagnosis	6 (11.3%)	42 (85.0%)	51.732	p < 0.001
Plan	Plan for Testing	23 (43.4%)	49 (98.0%)	33.912	p < 0.001
	Therapy/Treatment Plan	38 (71.7%)	50 (100.0%)	14.366	p < 0.001
	Referrals	9 (17.0%)	44 (87.0%)	49.146	p < 0.001
	Patient Education	8 (15.1%)	42 (85.0%)	46.185	p < 0.001
Overall Structure	Correct SOAP Sequence	18 (34.0%)	47 (94.0%)	37.295	p < 0.001

## Discussion

This audit demonstrated a substantial improvement in the quality of follow-up documentation at Hasahesa Teaching Hospital, with overall SOAP-sequence compliance increasing from 34.0% in Cycle 1 to 94.0% in Cycle 2. This dramatic improvement reflects the effectiveness of the intervention, which consisted of introducing a structured SOAP template supported by focused staff training. The results highlight the value of organized documentation in enhancing clinical accuracy, improving continuity of care, and promoting clearer communication among healthcare providers. However, further evaluation using direct clinical outcome indicators - such as complication rates or readmission trends - is needed to determine the broader clinical impact of these improvements.

Enhancements were observed across all assessed parameters, indicating that the intervention had wide-reaching effects on documentation practice and patient-safety culture. These results align with prior research demonstrating that structured documentation tools outperform unstructured, free-text approaches. For example, one study reported an increase in documentation quality from 64.35% to 77.2% after implementing standardized electronic templates, reinforcing the importance of structured formats in improving the usability and completeness of clinical records [7]. Similar improvements have been reported in paper-based environments, where training, combined with SOAP-based templates, resulted in a 56% rise in compliance with professional documentation standards [8].

Marked improvements were seen in the Subjective domain. Documentation of the chief complaint increased from 28 (52.8%) to 50 (100%), mirroring findings by Shirazi and Masood, who demonstrated that template-based approaches improve the accuracy and consistency of presenting-complaint documentation [9]. Documentation of the review of systems and past medical history rose from 12 (22.6%) to 44 (88%) and from 18 (34%) to 46 (92%), respectively. These areas are essential components of a comprehensive clinical assessment, and the improvements noted align with earlier studies linking systematic record-keeping to higher-quality patient histories [10].

One of the most important improvements was in medication and allergy documentation, which increased from 10 (18.9%) to 45 (90%). The low baseline rate highlighted a major gap in continuity of care and medication-safety practices. The striking improvement following the intervention underscores the role of structured templates in ensuring accurate medication lists - crucial for preventing adverse drug events, a leading cause of preventable inpatient harm [11].

Objective documentation also improved considerably. Although vital-sign documentation was already high at baseline (94.3%) and increased to 100%, physical-examination findings rose from 25 (47.2%) to 48 (96%), and lab/imaging documentation from 17 (32.1%) to 44 (89%). These gains indicate more consistent integration of diagnostic and examination data into follow-up notes. Previous research has shown that clinical deterioration is often preceded by subtle vital-sign changes that may go undocumented, especially respiratory parameters [12], making improvements in this category particularly relevant.

The Assessment and Plan domains also showed major gains. Primary diagnosis documentation increased from 26 (49.1%) to 50 (100%), and differential diagnoses from 6 (11.3%) to 42 (85%). Planning elements, such as investigation plans, treatment updates, referrals, and patient education, improved to between 85% and 100%, demonstrating stronger clinical reasoning and clearer decision-making in Cycle 2. These improvements likely reflect greater clinician engagement with structured formats and improved awareness of documentation expectations.

Overall, this audit provides strong evidence that structured SOAP templates, supported by targeted education, can significantly enhance documentation completeness in a resource-limited, paper-based setting. To sustain these improvements, structured documentation should be incorporated into departmental policy, orientation programs, and regular re-audit cycles. Future initiatives should assess whether improved documentation translates into measurable improvements in patient outcomes and clinical processes.

Limitations

Several limitations should be considered when interpreting these findings. First, this quality improvement audit was conducted in a single department within one hospital, which may limit its applicability to other settings with different staffing patterns or documentation workflows. Second, because all data were derived from paper-based records, the audit was influenced by common issues such as missing notes, inconsistent filing, and illegible handwriting. Third, the audit focused on documentation completeness rather than clinical accuracy, consistency, or quality; therefore, records classified as complete may still have contained clinical inaccuracies or insufficient detail. Fourth, the binary grading approach (complete/incomplete) did not capture variations in depth, clarity, or clinical reasoning within individual SOAP components. Fifth, the post-intervention assessment covered a three-month period, limiting conclusions regarding the long-term sustainability of the observed improvements. Finally, no patient-centered outcomes were assessed, and the relatively limited audit sample reflects the pragmatic nature of quality improvement work; therefore, while documentation improved substantially, its direct impact on clinical outcomes, patient safety, and care efficiency could not be determined.

## Conclusions

This clinical audit demonstrated that introducing a structured SOAP-based documentation template, supported by focused staff training and ongoing reinforcement, led to a clear and meaningful improvement in the quality and completeness of follow-up notes in the Internal Medicine Department at Hasahesa Teaching Hospital. The intervention improved the organization, clarity, and consistency of clinical documentation and supported a more systematic approach to daily patient review.

These findings underscore the value of simple, low-cost quality improvement strategies in strengthening documentation practices within resource-limited, paper-based healthcare settings. While the observed improvements were substantial, this audit was not designed to assess patient-centered outcomes, and the direct impact on clinical outcomes remains to be determined. Sustaining these gains will require continued staff education, routine monitoring, and formal integration of structured documentation tools into departmental policy. Future work should extend this approach to other clinical services and evaluate its influence on patient safety, continuity of care, and overall quality of service delivery.
